# Respiratory entrainment of units in the mouse parietal cortex depends on vigilance state

**DOI:** 10.1007/s00424-022-02727-2

**Published:** 2022-08-19

**Authors:** Felix Jung, Yevgenij Yanovsky, Jurij Brankačk, Adriano B. L. Tort, Andreas Draguhn

**Affiliations:** 1 Institute for Physiology and Pathophysiology, Heidelberg University, 69120 Heidelberg, Germany; 2 Department of Neuroscience, Karolinska Institutet, Stockholm, Sweden; 3 Brain Institute, Federal University of Rio Grande Do Norte, Natal, RN 59078‐900 Brazil

**Keywords:** Posterior parietal cortex, Unit firing, Respiration, θ rhythm, Sleep, Wakefulness

## Abstract

Synchronous oscillations are essential for coordinated activity in neuronal networks and, hence, for behavior and cognition. While most network oscillations are generated within the central nervous system, recent evidence shows that rhythmic body processes strongly influence activity patterns throughout the brain. A major factor is respiration (Resp), which entrains multiple brain regions at the mesoscopic (local field potential) and single-cell levels. However, it is largely unknown how such Resp-driven rhythms interact or compete with internal brain oscillations, especially those with similar frequency domains. In mice, Resp and theta (θ) oscillations have overlapping frequencies and co-occur in various brain regions. Here, we investigated the effects of Resp and θ on neuronal discharges in the mouse parietal cortex during four behavioral states which either show prominent θ (REM sleep and active waking (AW)) or lack significant θ (NREM sleep and waking immobility (WI)). We report a pronounced state-dependence of spike modulation by both rhythms. During REM sleep, θ effects on unit discharges dominate, while during AW, Resp has a larger influence, despite the concomitant presence of θ oscillations. In most states, unit modulation by θ or Resp increases with mean firing rate. The preferred timing of Resp-entrained discharges (inspiration versus expiration) varies between states, indicating state-specific and different underlying mechanisms. Our findings show that neurons in an associative cortex area are differentially and state-dependently modulated by two fundamentally different processes: brain-endogenous θ oscillations and rhythmic somatic feedback signals from Resp.

**Supplementary Information:**

The online version contains supplementary material available at 10.1007/s00424-022-02727-2.

## Introduction

Neuronal network oscillations are important for coordinating multi-neuronal activity patterns within and across different brain regions [16]. Multiple lines of evidence show that such coordinated patterns, in turn, are a key mechanism underlying cognition and behavior [21, 22, 29]. While most oscillation patterns result from brain-endogenous network dynamics, there is growing evidence that they can also be generated or modulated by feedback from rhythmic activity in the body. Such rhythmic feedback signals include respiration (Resp) [28, 34, 62], cardiac activity [14, 30, 66], and the gastric rhythm [50, 51]. Modulation of neuronal behavior occurs across species, including humans [31, 70], and it affects widespread regions of the brain [52, 62]. Resp-driven oscillations are not only visible at the network level but also do modulate spiking of single neurons in different regions including frontal cortex [6, 40], dentate gyrus [13], and parietal cortex [37]. In line with these findings, several lines of evidence show that oscillating somatic feedback signals affect cognition in humans [46, 70] and rodents [4, 28].

Neuronal network oscillations cover a large range of frequency bands, and different oscillation patterns occur in different functional states of the brain which, in turn, reflect specific cognitive-behavioral states of the organism [16]. This raises the question of how brain-endogenous and somatically generated oscillations co-occur and interact. A recent example illustrates the complexity of such interactions: brain-endogenous theta (θ, 5–12 Hz) strongly modulate local gamma (γ, 40–160 Hz) oscillations, a fundamental pattern for cortical computation and cognition [7, 11, 21, 42, 46, 60, 61]. Recordings from mice have shown that the prominent θ-γ-coupling during REM sleep [53] is modulated by Resp rate, expressing maximum coupling at a moderate Resp frequency of around 5 Hz [25]. This modulation may be associated with an intermediate level of arousal which is reflected by breathing frequency during REM sleep [59, 68].

Thus, brain-endogenous and body rhythms show complex, state-dependent interactions which are likely to be functionally relevant. It is therefore important to untangle the effects of different oscillations on neuronal discharges, especially in regions where oscillations with overlapping frequency bands co-occur. This is the case for θ- and respiration-related oscillations (RR) in cortical networks of the mouse [13, 69]. Here, we studied the impact of both oscillation patterns on neuronal discharge behavior in the posterior parietal cortex, a multi-modal associative area with known modulation by both θ [56] and Resp [37]. In our previous research, we investigated the influence of θ and Resp with a minimal frequency overlap in parietal networks focusing on two vigilance states with either prominent θ (REM sleep) or in the absence of θ (waking immobility, WI) [32]. In the present study, we aimed to extend our past findings by systematically comparing the impact of Resp and θ on unit firing regardless of frequency overlap in active waking (AW) during sniffing and prominent θ oscillation. In addition, we asked the question of how Resp influences unit firing in the other sleep state without θ but with prominent slow-wave activity (non-REM sleep, NREM). Thus, we recorded units and local field potential (LFP) activity using chronically implanted tetrodes in the posterior parietal cortex of mice. As a reference signal for Resp, we measured rhythmic pressure fluctuations in a plethysmograph, which is more stable than the fluctuating field potentials reflecting RR [30]. Our results revealed a strong, state-dependent modulation of unit discharges by both θ and Resp. Importantly, our results during AW showed a disparity between the observed power of LFP oscillations (dominated by θ) and the proportion of modulated units by either θ or Resp, arguing for a careful interpretation of mesoscopic activity signals such as the LFP compared to unit activity.

## Material and methods

For ethical statement on animal experiments, please see the “Declarations” section below. Parts of the data recorded during REM sleep and WI have been previously published in a different context [37].

### Animal care and housing

Mice of the C57BL/6 N strain were obtained from Charles River and housed inside a ventilated Scantainer with an inverted 12/12-h light–dark cycle (light on at 8:00 p.m.) and free access to water and food. In all experiments, male animals were used due to their higher availability and because sex differences were not expected for the present study.

### Surgery and electrode implantation

A total of thirteen male mice weighing 23–30 g (13–21 weeks old) were anesthetized with a mixture of isoflurane and medical oxygen (4% isoflurane for induction, 1.5–2.5% for maintenance). In 10 animals, between 5 and 7 tetrodes were chronically implanted into the right and left parietal cortex at various depths (2 mm posterior bregma, 1.5 mm lateral, 0 to 0.8 mm ventral). In three animals, 16-channel silicon probes (A1 × 16-3 mm-50–177-CM16LP; NeuroNexus Technologies) were chronically implanted into the right parietal cortex. Inter-electrode distance was 50 µm, and implantation was perpendicular to the cortical surface such that the uppermost electrode was located superficially, the lowest at 750 µm). For further details, see [37].

### Electrophysiology in freely moving mice

After 1 week of recovery, the animals were habituated to a whole-body plethysmograph (EMKA Technologies, S.A.S., France) [25, 35] which was adapted for simultaneous recording of Resp and brain electrophysiology. Although RR are detectable in the parietal cortex [62], they vary strongly depending on vigilance states, Resp frequency [24, 35], and other hitherto unknown factors. To avoid signal instabilities due to this variability of RR, we used the Resp signal instead, which is directly derived from Resp-induced pressure changes in the plethysmograph (see Methods) and remains stable throughout all vigilance states. Movements were detected with 3-D accelerometry. Animals were recorded in several sessions of up to 4 h on consecutive days.

### Behavioral staging

Artifact-free periods of recorded potentials were visually identified. Animals with linear silicon probes had excessive amounts of movement artifacts during AW. We therefore limited analysis of this particular state to the remaining 10 animals with implanted tetrodes. Behavioral and vigilance states were assessed according to motion/immobility (based on accelerometer activity) as well as the LFP dynamics in the posterior parietal cortex. In short, AW was identified by high accelerometer activity, continuing high Resp rate (sniffing), and θ oscillations. In contrast, REM sleep was characterized by absent or very low accelerometer activity and the presence of regular θ oscillations which followed slow-wave sleep and terminated with the animal’s awakening. NREM sleep was characterized by low frequency Resp, the absence of both accelerometer activity and θ oscillations, and by the presence of prominent slow-wave activity. WI was characterized by the absence of motion (i.e., accelerometer activity) and of θ or slow-wave activity. The beginning and end of behavioral states were manually marked using a custom-made graphical user interface programmed in MATLAB. Epochs were subsequently concatenated for each recording session.

### Data analysis

Built-in and custom-written routines in MATLAB (The MathWorks) were used for data analysis. For analysis of power spectral density, we used the *pwelch.m* function (50% overlap, 4-s Hamming windows). Spike detection, sorting, and quality assessment were done using the sorting algorithm “*Wave_clus*” [12, 49], similar to [37]. Units with fewer than 100 spikes during the total recording time were excluded. Four quality tests were applied to the initial results of sorting: 1) the Hill test [32]: < 1% of detected spikes occur within the first 1 ms after a spike; 2) isolation distance [26], which estimates the distance of each cluster to other clusters (> 15); 3) cluster quality L_ratio_ (< 0.25). L specifies the degree of separation between two clusters, a low value of L indicates that the cluster is well separated. However, clusters may be of different size and L divided by the number of spikes (*L*_ratio_) allows larger clusters to tolerate more contamination [54]; 4) correlation coefficient (*r* < 0.98) between spike waveforms for each unit across all recording sites (four for tetrodes and three for probes). *Firing rates:* Depending on behavioral state, units may fire more in bursts or may be silent for long periods. Silent periods are significantly longer during sleep compared to waking. The percentage of spike intervals within a burst (≤ 5 ms) differs significantly among behavioral states (Supplemental Fig. 1). In order not to distort the estimation of regular firing rates and to compare among behavioral states, long periods of non-spiking and burst firing (spike interval ≤ 5 ms) were excluded from the calculation of firing rates for each unit. Periods of non-firing were found by identifying groups of spikes (minimal 2 spikes per group) throughout the entire range of spike times with an initial threshold for inter-group intervals of ≤ 0.1 s. With threshold increments of 0.1 s, further groups were included until the total number of spikes was reached. Inter-spike intervals exceeding the upper threshold defining all spike groups were identified as silent periods and excluded from the calculation of firing rate. *Spike-field coupling:* phase time series were obtained by using the *hilbert.m* function (for details, see [37]). Spike-phase distributions were then calculated by relating the spike times with the instantaneous phases of the oscillating field potential. The *Rayleigh test* for uniformity of circular distributions was used to check for statistical significances. Coupling strength to Resp and θ was estimated using the circular mean resultant length (R). An overview of all units for individual animals and behavioral states is provided in Table 1.

**Fig. 1:**
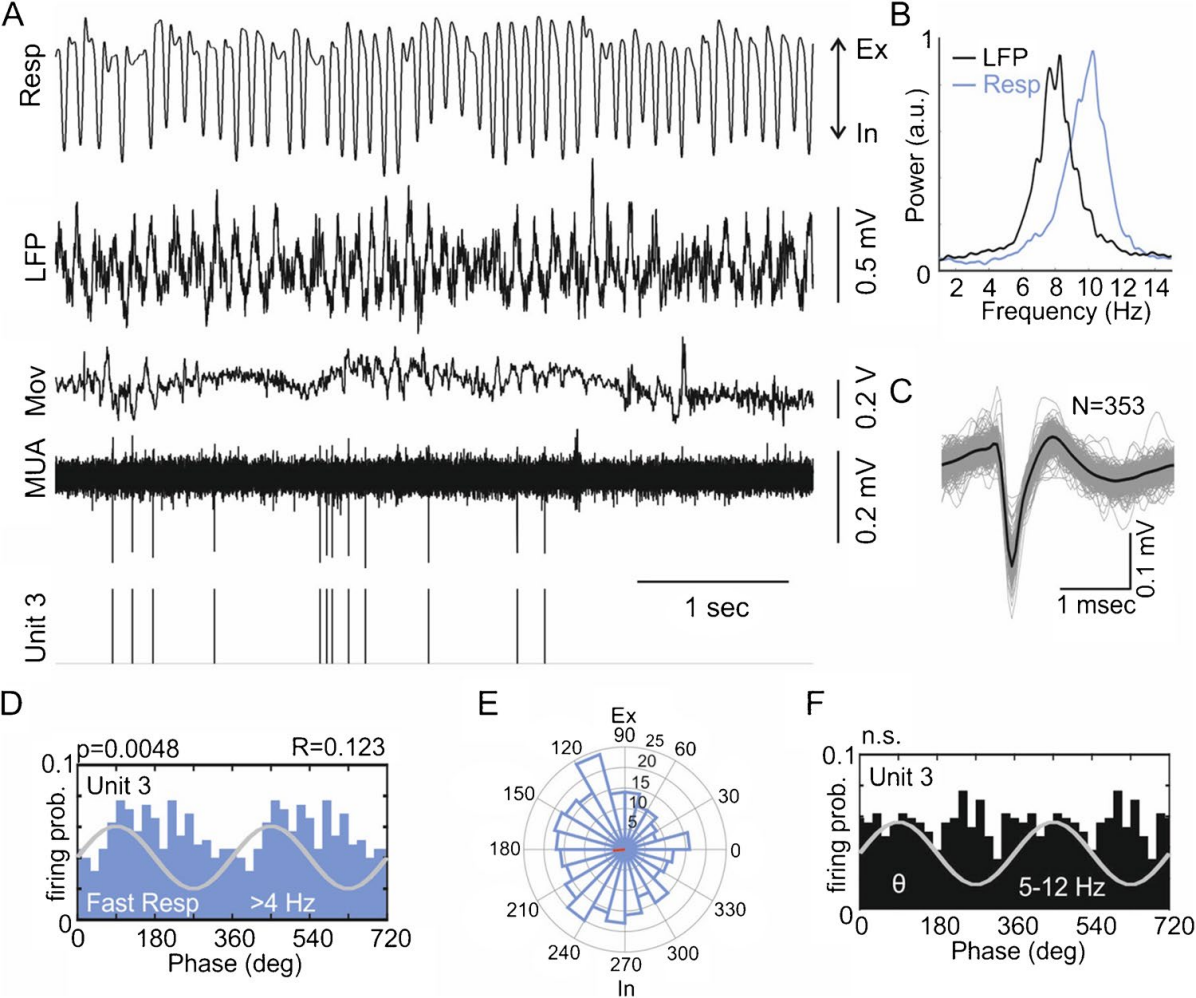
Parietal cortex unit modulated by fast respiration during active waking. **A**: Raw respiration (Resp) signal, local field potential (LFP) from the parietal cortex, accelerometer activity indicating movement (Mov), multi-unit activity (MUA) from one tetrode wire, and the time stamps of a sorted and quality tested unit (unit 3) during active waking. **B**: Power spectra of the parietal cortex LFP (black) and Resp (blue) during active waking. θ and Resp are partly overlapping, yet with different frequencies of maximal power (Resp > θ). **C**: Averaged unit waveform (black) on the background of superimposed 353 single spikes (gray). **D**: Phase histogram of unit 3 firing probability based on Resp cycles (frequency range 4–14 Hz; “fast respiration”) showing significant modulation of unit discharges. R indicates coupling strength. **E**: Polar plot showing Resp phase-dependent activation of unit 3. The firing probability is maximal between 90 (maximum of expiration, Ex) and 270 degrees (maximum of inspiration, In). **F**: Phase histogram of unit 3 based on θ cycles; the unit was not significantly modulated by θ (5–12 Hz)

**Table 1:** List of units

List of units for individual animals and each behavioral state																
State	REM sleep					NREM sleep			Wake Immobility			Active Waking				
Animal	#	N	R	θ	B	#	N	R	#	N	R	#	N	R	θ	B
VW01	19	15	1	3	0	49	41	8	19	16	3	38	13	21	3	1
VW02	12	8	3	1	0	2	1	1	3	2	1	34	12	5	9	8
VW03	21	15	3	2	1	12	6	6	3	3	0	12	7	4	1	0
VW04	20	14	2	4	0	7	6	1	3	3	0	24	11	12	1	0
VW05	69	51	8	9	1	19	18	1	39	29	10	26	22	2	2	0
VW06	36	8	0	28	0	8	7	1	15	8	7	43	15	13	9	6
VW07	31	12	3	11	5	16	14	2	9	6	3	15	4	8	0	3
VW08	48	21	2	19	6	8	7	1	9	8	1	15	9	3	1	2
VW10	35	21	9	4	1	11	10	1	6	6	0	40	16	15	2	7
VW11	52	32	3	16	1	57	48	9	14	12	2	20	11	7	1	1
G01	99	51	5	33	10	115	92	23	67	60	7	N/A (probe recordings)				
G03	24	14	2	6	2	46	42	4	8	7	1	N/A (probe recordings)				
G04	105	64	14	18	9	123	95	28	37	33	4	N/A (probe recordings)				
Total	571	326	55	154	36	473	387	86	232	193	39	267	120	90	29	28
Mean	43.9	26.1	4.2	11.8	2.8	36.4	29.8	6.6	17.8	14.8	3	26.7	12	9	2.9	2.8

*#* number of units, *N* not modulated, *R* modulated by respiration, *θ* modulated by theta, *B* modulated by both theta and respiration

### Histology

After the last experiment, the animals were deeply anaesthetized with ketamine-xylazine (30 mg/kg + xylazine 10 mg/kg) and perfused transcardially with PBS followed by 4% paraformaldehyde. The position of tetrodes and multichannel probes was then verified by fluorescence (staining with DAPI: 4,6-Diamidin-2-phenylindol) and fluorescence microscopy. For further details, see [37].

### Statistics

Data are presented as means ± SEM for Gaussian distributions or as medians ± 25/75% percentiles for non-Gaussian distributions. The Mann–Whitney* U* test was applied for comparing coupling strength. For comparisons of proportions, the Chi-squared test was used. Two-sample testing of circular data was performed with the Watson’s U2 test. *p* values of < 0.05 were considered as statistically significant.

## Results

We recorded field potentials and Resp from 13 freely behaving male mice (see Methods). In line with previous publications [8, 62, 71], we found a strong state-dependence of θ oscillations and slow-wave activity in the posterior parietal cortex: During REM sleep and AW, LFPs were dominated by θ. In contrast, high voltage slow waves were characteristic during NREM sleep. During WI, both slow waves and θ were absent.

### Unit firing is modulated by respiration

The presence of RR in the parietal cortex depends on behavioral states [62] and varies strongly with Resp frequency [35] and probably further factors [24]. We therefore used the Resp signal from plethysmography which could be reliably recorded during all four behavioral or vigilance states (see Methods). Figure 1A shows an example recording during AW with Resp rates exceeding and partly overlapping the frequency of θ in the LFP (see power spectral densities in Fig. 1B). In this example, we illustrate the detection, waveform, and rhythmic modulation of an identified unit (Fig. 1C–F). This unit is significantly coupled to Resp when the breathing frequency is > 4 Hz (fast Resp, coupling strength *R* = 0.123, Fig. 1D). In this situation, firing probability is highest between maxima of expiration (Ex, 90 deg) and inspiration (In, 270 deg, Fig. 1E). At the same time, this example unit is not modulated by θ (5–15 Hz, Fig. 1F; for modulation by slow Resp, see below). Similar analyses were applied to all recorded units in all animals and states (*n* = 1543, see Table 1), giving a systematic account of state-dependent unit entrainment by θ and Resp, as described below (for further details and examples, see Supplemental Figs. 2 and 3).

**Fig. 2:**
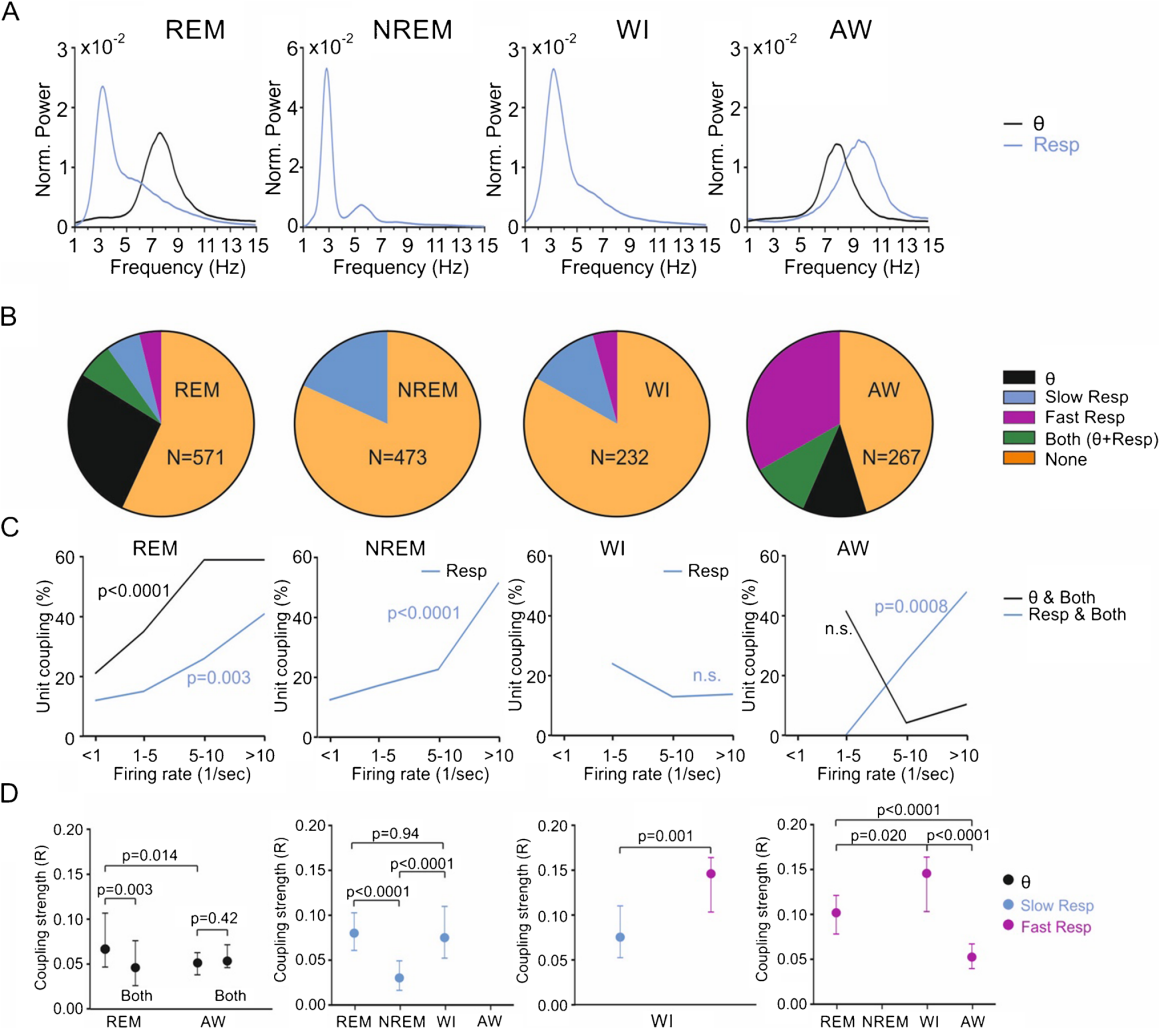
Modulation of parietal cortex units by θ and respiration in sleep and wakefulness. **A**: Mean power of slow rhythms (Resp, blue; θ, black) during REM sleep, NREM sleep, waking immobility (WI) and active waking (AW). Note absence of θ activity in NREM sleep and WI. **B**: Proportion of units modulated by θ (black), slow Resp (blue), fast Resp (magenta) or θ and Resp (slow and fast) simultaneously (“Both,” green). Orange indicates non-modulated units. Note that θ-modulation dominates in REM sleep whereas modulation by fast Resp prevails in AW. **C**: Relation between firing rate and unit coupling to θ or Resp. Coupling to θ increases significantly with firing rate in REM but not in AW. Unit modulation by Resp increases significantly with firing rate in REM, NREM and AW but does not change in WI (see Table 2 for details). **D**: Coupling strength of units to θ (black dots and errors: 25% and 75% percentiles) and respiration (blue: slow Resp, magenta: fast Resp). Differences in coupling strength for the respective states are indicated by brackets and *p *values

**Fig. 3:**
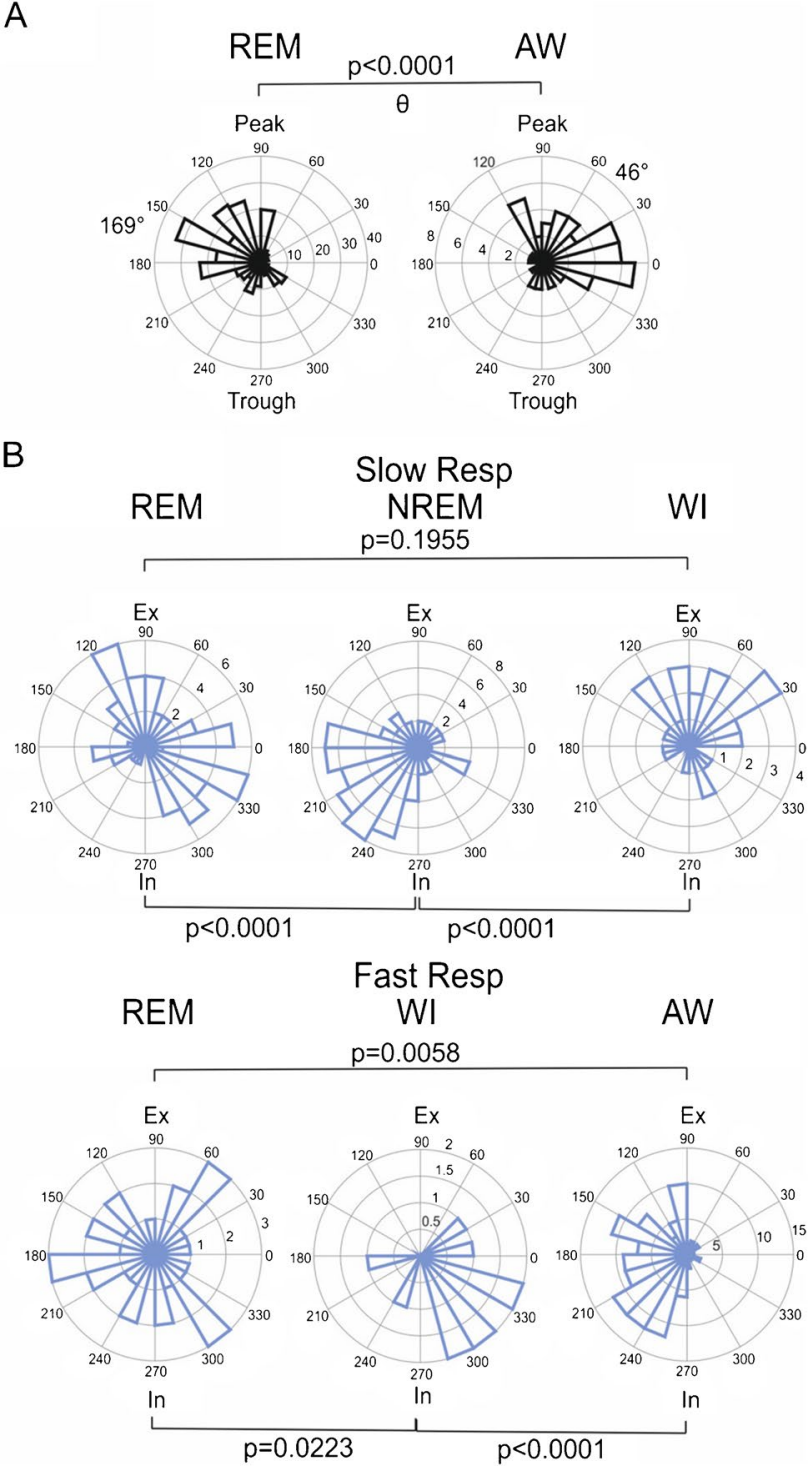
State-dependent phase preference of parietal cortex. **A:** Units modulated by θ have significantly different θ phase preferences in REM (169 deg, after peak) compared to AW (46 deg, before peak). **B**: Units coupled to slow Resp are entrained to different phases of respiration in REM compared to NREM and in NREM compared to WI; units in NREM preferentially fire during the rising flank of inspiration (In, 180 to 270 deg), whereas units in WI fire mostly during expiration (Ex, 0 to 180 deg). In AW, units modulated by fast Resp (fR) preferentially fire during the rising flank of In and the falling flank of Ex. *p* values are based on the Watson U2 test

### Unit coupling depends on vigilance state

The power spectral peaks of the LFP and Resp signals were largely overlapping at the θ range in AW (Fig. 2A right panel) and partially overlapping in REM (Fig. 2A left panel), whereas θ was absent in NREM and WI (Fig. 2A middle panels). Depending on vigilance state, the animal’s breathing frequency varied strongly between 1 and 14 Hz. For further analysis, we differentiated between slow (1–4 Hz) and fast (> 4 Hz) Resp. The 4 Hz threshold corresponds to boundaries in the spectral distribution of Resp frequency in each behavioral state (see Supplemental Fig. 4). Both slow and fast Resp were present in REM and WI. In contrast, NREM contained exclusively slow Resp, and AW almost exclusively fast Resp. The percentage of units modulated by θ, slow Resp, fast Resp or by both θ and Resp simultaneously (referred to as “both” units) varied among vigilance states (Fig. 2B). As expected, the percentage of units modulated by any of the slow rhythms (θ and Resp, pooled) was significantly higher in states with θ present compared to non-θ states (AW = 54.7%; REM = 43.1%; NREM = 18.2%; WI = 16.8%; see Table 2 for details). Moreover, the percentage of units coupled to Resp (AW = 33.3%; REM = 9.8%) and both Resp and θ simultaneously (AW = 10.1%; REM = 6.3%) was larger in AW compared to REM. Interestingly, despite the presence of θ in both states, significantly more units were modulated by θ in REM (27.0%) compared to AW (11.2%). Finally, coupling percentage to Resp was not different between WI (16.8%) and NREM (18.2%). In summary, the coupling of units to θ or Resp is highly state-dependent. More units are modulated by θ in REM whereas more units are modulated by Resp in AW.

**Fig. 4:**
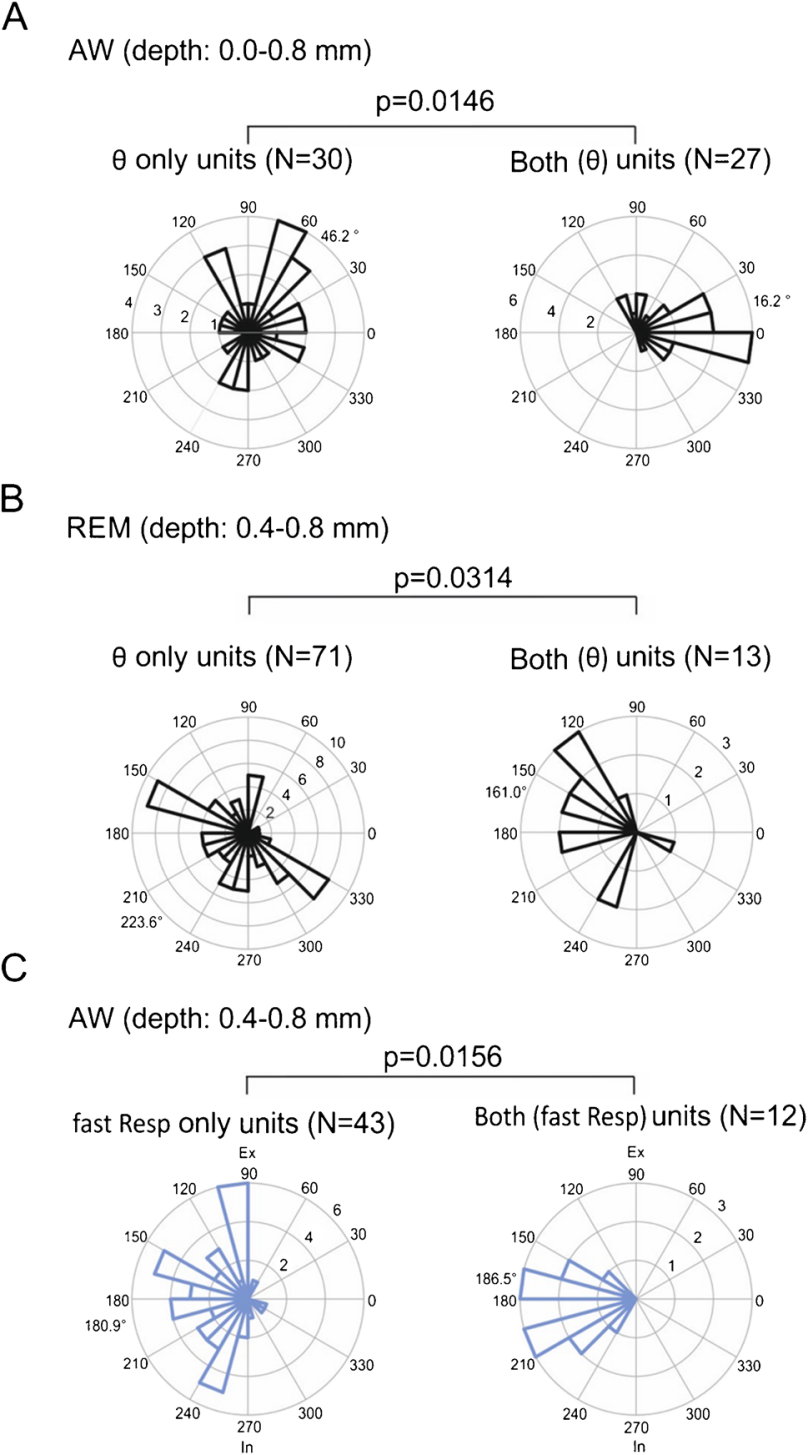
State- and depth-dependent interference of Resp and θ on spike phase preference. **A:** θ-only modulated units have a significantly different θ phase preference than units co-modulated by Resp during AW. **B:** θ-only modulated units have a significantly different θ phase preference than units co-modulated by Resp in deep layers (0.4–0.8 mm depth) during REM. **C:** Fast Resp-only modulated units have a significantly different Resp phase preference than units co-modulated by θ in superficial layers (< 0.4 mm depth) during AW. *p* values are based on the Watson U2 test

**Table 2:** Details of unit behavior

Percentage of units with coupling to θ or Resp							
*Unit type*				*Property*	*Test*		*p*
All except None				θ-states > non-θ states	Chi-square		< 0.0001
All except None				REM < AW	Chi-square		0.0017
θ + Both				REM > AW	Chi-square		0.0007
Resp + Both				REM < AW	Chi-square		< 0.0001
Resp + Both				WI = NREM	Chi-square		0.6541
Dependence of coupling on firing rate							
*Unit type*	*State*		*Property*		*Test*		*p*
θ + Both	REM		**↑** with firing rate		Chi-square		< 0.0001
θ + Both	AW		no change		Chi-square		0.9971
Resp + Both	REM		**↑** with firing rate		Chi-square		0.003
Resp only	NREM		**↑** with firing rate		Chi-square		< 0.0001
Resp only	AW		**↑** with firing rate		Chi-square		0.0008
Resp only	WI		no change		Chi-square		0.1052
Coupling strength: θ versus Resp							
*Unit type*		*Rhythm*		*Property*	*Test*		*p*
θ only		θ		REM > AW	Mann–Whitney		0.0136
Both		θ		REM = AW	Mann–Whitney		0.9476
sR only		Resp		REM > NREM	Dunn’s multiple comparison		< 0.0001
sR only		Resp		REM = WI	Dunn’s multiple comparison		> 0.9999
sR only		Resp		NREM < WI	Dunn’s multiple comparison		< 0.0001
fR only		Resp		REM > AW	Dunn’s multiple comparison		< 0.0001
fR only		Resp		WI > AW	Dunn’s multiple comparison		< 0.0001
fR only		Resp		REM = WI	Dunn’s multiple comparison		0.4658
*State*		*Rhythm*		*Property*	*Test*		*p*
REM		θ		θ only > Both	Mann–Whitney		0.0028
WI		Resp		sR only < fR only	Mann–Whitney		0.0013
REM		Resp		sR only = fR only	Mann–Whitney		0.9421
Phase preference							
*Unit type*				*Property*	*Test*		*p*
θ + Both				REM vs. AW/ different	Watson U^2^		< 0.0001
sR only				REM vs. NREM/ different	Watson U^2^		< 0.0001
sR only				NREM vs. WI/ different	Watson U^2^		< 0.0001
sR only				REM vs. WI/ not changed	Watson U^2^		0.1955
fR only				REM vs. WI/ different	Watson U^2^		0.0223
fR only				REM vs. AW/ different	Watson U^2^		0.0058
fR only				WI vs. AW/ different	Watson U^2^		< 0.0001
Phase preference in Both units							
*State (depth)*				*Property*	*Test*	*p*	
AW (all)				θ only vs. Both/ different	Watson U^2^	0.0146	
AW (deep)				fR only vs. Both/ different	Watson U^2^	0.0156	
REM (deep)				θ only vs. Both/ different	Watson U^2^	0.0314	

*AW* active waking; *WI* wake immobility; *NREM* non-REM sleep; *REM* REM sleep; *both* units modulated by both rhythms (θ and respiration) simultaneously; *θ* units modulated by θ; *Resp* units modulated by respiration; *fR* units modulated by fast respiration; *sR* units modulated by slow respiration; *None* units not modulated, neither by θ nor by respiration; *all* units in all layers (0–0.8 mm); deep: units in deeper layers (0.4–0.8 mm)

### Unit coupling depends on firing rate

Next we analyzed whether unit coupling to θ or Resp was influenced by the mean firing rate (FR). For estimation of FR, bursts and spiking gaps were excluded (see Methods). We found that the percentage of units coupled to θ or Resp varied with FR in a state-dependent manner. During REM, coupling percentage to θ increased with increasing FR (Fig. 2C left, see details in Table 2); in contrast, during AW, the percentage of coupled units showed a non-significant trend to decrease (Fig. 2C right). Coupling to Resp increased with FR in REM, NREM, and AW but not in WI (Fig. 2C and Table 2). In conclusion, unit coupling to either rhythm varies with FR, but this correlation depends on behavioral or vigilance state.

### Coupling strength depends on vigilance state

We next measured the strength of coupling between individual units and the underlying oscillations. Table 2 shows the outcome of statistical tests for the most relevant comparisons. We found that coupling strength for units exclusively coupled to θ (and not Resp) was higher in REM compared to AW (Fig. 2D left). Coupling strength to slow Resp was lower in NREM compared to REM and WI (Fig. 2D middle left). In WI, coupling strength to fast Resp was higher than to slow Resp (Fig. 2D middle right). Coupling strength to fast Resp was highest in WI compared to REM and lowest in AW (Fig. 2D right). In all, we conclude that coupling strength differs among vigilance states, between the two types of slow rhythms, and also with Resp frequency.

### Unit firing and phase preference

Finally, we looked for phase preference of units modulated by θ or Resp and found pronounced state-specific differences. Preference to θ phase differed between REM and AW (Fig. 3A). In AW, θ units fired preferentially at the rising flank of θ (~ 46 deg), whereas in REM they fired after the peak (~ 169 deg). Preference for either inspiration or expiration in units coupled to slow Resp was different in NREM compared to REM and WI (Fig. 3B, upper row). For units modulated by fast Resp, Resp phase preference differed in all three states: REM, WI, and AW (Fig. 3B, lower row). Interestingly, units exclusively coupled to θ had a different phase preference than units coupled to θ and Resp simultaneously (Both units) in all cortical layers for AW (Fig. 4A) and in deep layers for REM (Fig. 4B). Phase preference for fast Resp in Both units differed only in deep layers for AW (Fig. 4C). For statistics, see Table 2, and for a systematic overview of depth-dependent influences on phase preferences, see Supplemental Tables 1 and 2.

## Discussion

Our data shows specific and robust modulation of units in the parietal cortex by both Resp and θ. However, the rhythmic entrainment of units by either of the two slow oscillations depends strongly on (i) vigilance state and (ii) mean firing rate (FR). In order to gain a systematic overview, we compared all major sleep and waking states. In two of these states, AW and REM sleep, both oscillations are simultaneously present. At the field potential level, θ oscillations are dominant in both states (see [37]). At the cellular (unit) level, however, entrainment by Resp is prevailing during AW while θ is more efficient during REM sleep. This observation emphasizes potential discrepancies between the observed power of network-level oscillations and behavior of individual neurons which should be kept in mind when interpreting collective neuronal signals (field potentials, EEG, MEG). A second finding of the present study is that in several states, coupling of units to θ or Resp was dependent on discharge frequency. Whether this activity-dependence of entrainment reflects differences in network integration of different cell types or of differentially active neurons of the same type is presently unclear. In any case, the correlation between neuronal activity and coupling to slow network oscillations may be important for understanding ensemble activity in oscillating networks [17, 19]. A further new observation is that units couple to different phases of Resp depending on the behavioral state. This finding may be explained by state-dependent differences in Resp-related synaptic input to the parietal network, by recruitment of different types of interneurons within the parietal cortex, or by other, more complex state-dependent interactions between different networks. In any case, all findings underline the strong state-dependence of unit entrainment by both Resp and θ oscillations.

Some of the present findings from freely moving mice confirm our earlier results from head-fixed [13] and urethane-anaesthetized animals [69]. In these preparations, we had already reported Resp-driven modulation of hippocampal [62, 69] and posterior parietal and prelimbic neurons [37, 71]. While our previous observations were mainly done in REM sleep or wake immobility, we here provide a systematic comparison of modulation by θ and Resp during all major states of vigilance/behavior: REM sleep, NREM sleep, WI, and AW. The latter is of particular interest since it contains θ oscillations and, at the same time, strong Resp-coupled inputs from sniffing behavior. In this state, entrainment of neurons by Resp was dominant, with particularly strong effects on neurons with high discharge frequency. It is well feasible that this reflects the integration of sensory signals from nasal Resp or sniffing in the multimodal association network of the parietal cortex.

Earlier reports have shown that neurons in several neocortical regions, including the parietal cortex of rats and mice, are modulated by theta oscillations during locomotion and REM sleep [56]. Here, we confirm this finding for REM sleep, while foraging behavior could not be tested within the limited space inside the plethysmograph. Interestingly, our results show that during REM sleep parietal cortex units modulated by θ preferentially couple after the peak of this oscillation. In contrast, units fire more before the θ peak during AW. Previous studies showed no phase difference for θ-modulation between locomotion and REM sleep [56] in parietal networks. This discrepancy could be due to our recordings taking place in a spatially confined environment (plethysmograph), while the animals in previous studies could display more elaborate locomotor activity. In addition, AW in our study included sniffing and θ oscillations.

We did not observe an effect of cortical depth on the θ phase-modulation of unit firing during AW, but, intriguingly, the preferred θ phase shifted between deep and superficial layers during REM sleep. Akin to this result, in the visual cortex, phase-coupling to θ oscillations in layers 5 and 6 differs from layers 2–4 [20]. The putative interaction and competition of θ oscillations and Resp are topics of ongoing research. In the present study, we found that units coupling exclusively to θ display different preferred θ phases from units co-modulated by Resp in AW. We found a similar tendency during REM sleep. Strikingly, this difference is most prominent in the superficial layers in AW and in the deep layers in REM sleep. These results could indicate distinct, state-dependent mechanisms of Resp transmission (and unit entrainment) in parietal networks.

With regard to unit modulation by Resp, a more complex picture emerged. Across most of the behavioral states, we found a tendency of phase-modulation during inhalation, confirming previous reports investigating other neocortical networks, including the orbital [40], prefrontal [6, 38], and visual areas [38]. However, our results also illustrate striking state- and breathing frequency-depending contrasts. As such, we found that during WI phase-modulation to the inhalation phase of fast Resp is dominant while shifting towards the exhalation phase during slow Resp. The neurophysiological underpinnings of these findings and the state-dependent transmission of Resp signals are yet to be identified.

In contrast to θ oscillations which are thought to be volume conducted from the hippocampus [23], it is unclear how Resp-entrained signals are transmitted to distant cortical brain areas. Clearly, feedback from the nasal airstream plays a role, since RR diminishes after tracheotomy [69], bulbectomy [4, 6], chemogenetic inhibition of the OB [43], depletion of the olfactory epithelium [38, 44], or nasal occlusion [44]. It may well be that mechanical stimulation of olfactory epithelial cells by airflow [1] is critical for RR generation [58]. However, Karalis and Sirota [38] recently demonstrated that lesioning the olfactory epithelium abolishes RR at the field potential level but does not eliminate neuronal entrainment by Resp, arguing for additional non-nasal sources of Resp-related activity modulation, possibly by collateral discharges from the rhythm-generating respiratory networks in the brainstem. An additional possibility is direct mechanical effects on neuronal activity. Cortical neurons do react to weak mechanical stimulation [47], and mechanosensitive Piezo2 ion channels have recently been shown to be present in cortical pyramidal cells, opening the possibility that minor pressure fluctuations in the brain parenchyma may translate into rhythmic entrainment of activity [3, 15]. Mechanical transduction processes could likewise mediate the heartbeat-dependent modulation of activity [14, 30, 66] for which there is no central rhythm generator. Additionally, increased cerebral blood flow was found to occur during REM sleep [63] modulating the power of fast gamma oscillations (80–110 Hz) [5], which highlights the putative role of blood flow-mediated mechanical transduction as a factor in mediating LFP signals. Our present results on modulation of neuronal activity by respiration do not allow to distinguish between the different possible mechanisms which are, notably, not mutually exclusive. In any case, we confirm that the power of the LFP is not consistently correlated with the strength of unit entrainment [38] suggesting that lamina-specific synaptic input is not the only mechanism of respiration-entrained neuronal discharges. Which of the two other mechanisms (corollary discharges from brainstem rhythm generators or mechanical stimulation of pyramidal cells) is responsible for the observed modulation of neuronal activity remains presently unclear.

Resp-related network activity is a brain-wide phenomenon [62], and respiratory modulation of unit activity was demonstrated in a large number of brain regions [4, 6, 13, 37, 38, 40, 69]. This poses the question of its putative role for brain function. It has been suggested that the brain-wide coordination of activity by slow network oscillations contributes to signal integration between different neuronal networks [16, 33, 36, 56]. RR may provide such a synchronizing signal, independent from their immediate relation to Resp or olfaction. The parietal cortex was found to play a critical role in decision-making processes [2, 27, 41, 45, 48] that strongly rely on the brain-wide integration of sensory information, behavioral and internal state and intended actions. It seems possible that RR provides a temporal scaffold for the underlying computations in the parietal cortex. Moreover, the parietal cortex serves important roles in spatial navigation [27, 41, 67]. Whether spatial cognition is specifically modulated by Resp is currently unknown, but would be well compatible with our present findings, especially the state-dependent expression of RR and its coordination with θ oscillations. A closely related cognitive process is spatial or declarative memory formation [10]. Of note, respiratory signals modulate hippocampal sharp-wave ripples [38, 43]—a biomarker of memory consolidation [9]—implicating a role of Resp in the underlying processes [18, 28]. Importantly, the presence of localized, concurrent ripple oscillations in the parietal cortex was recently observed in rats [39] possibly aiding information transfer from hippocampal to neocortical networks during memory consolidation. Interestingly, a recent study by Tingley and colleagues [57] found that hippocampal sharp wave-ripples additionally influence metabolic processes, highlighting the embeddedness of brain function within the whole body [55, 64] in which respiratory signals potentially serve a critical role [65].

Taken together, accumulating evidence shows the impact of bodily signals such as Resp on brain dynamics, opening possibilities to formulate and test novel hypotheses on the interaction between neuronal activity, behavior, and cognition. In addition, our present findings underline the state-dependence of entrainment of neocortical neurons, which can be driven by θ, Resp, or both rhythms depending on firing rate and behavioral state.

## Supplementary Information

Below is the link to the electronic supplementary material.

Supplementary file1 (DOCX 873 kb)

## Data Availability

The datasets generated during and/or analyzed during the current study are available from the corresponding author on reasonable request.
