# Supplementary material for: Respiratory entrainment of units in the mouse parietal cortex depends on vigilance state
Source: Pflugers Arch. 2022 Aug 19;475(1):65–76. doi: 10.1007/s00424-022-02727-2 (PMC9816213; doi:10.1007/s00424-022-02727-2)
Supplement: Supplementary file 1 — Supplementary file1 (DOCX 873 kb) [file 424_2022_2727_MOESM1_ESM.docx]

**Supplemental Material (Jung et al. )**

**
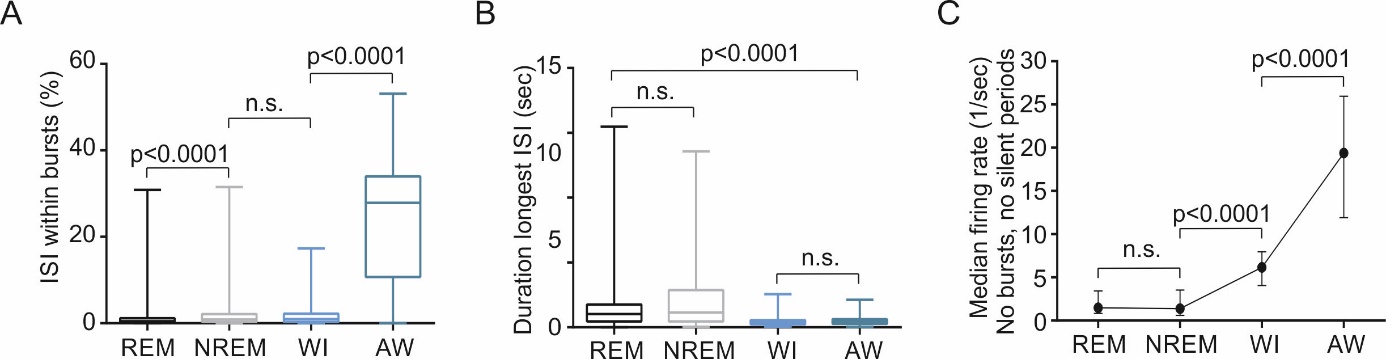
**

**Supplemental Figure 1:** **Spike bursting and longest inter-spike intervals depend on behavior. A:** Percentage of inter-spike intervals (ISI) falling within a spike burst (defined as ISI ≤ 5 ms) significantly differs among behavioral states (REM, N=571 units; NREM, N=473 units; WI, N=232 units; AW, N=267 units; Kruskal-Wallis test: p<0.0001, corrected for multiple comparisons using Dunn’s test: REM vs NREM: p<0.0001; REM vs WI: p<0.0001; NREM vs. WI: n.s., WI vs. AW: p<0.0001). **B:** Duration of longest inter-spike intervals significantly differs between sleep (REM and NREM) and waking (WI, AW) but not within sleep or waking states (same statistical test as in A). **C:** Median firing rates without burst and silent periods.

**
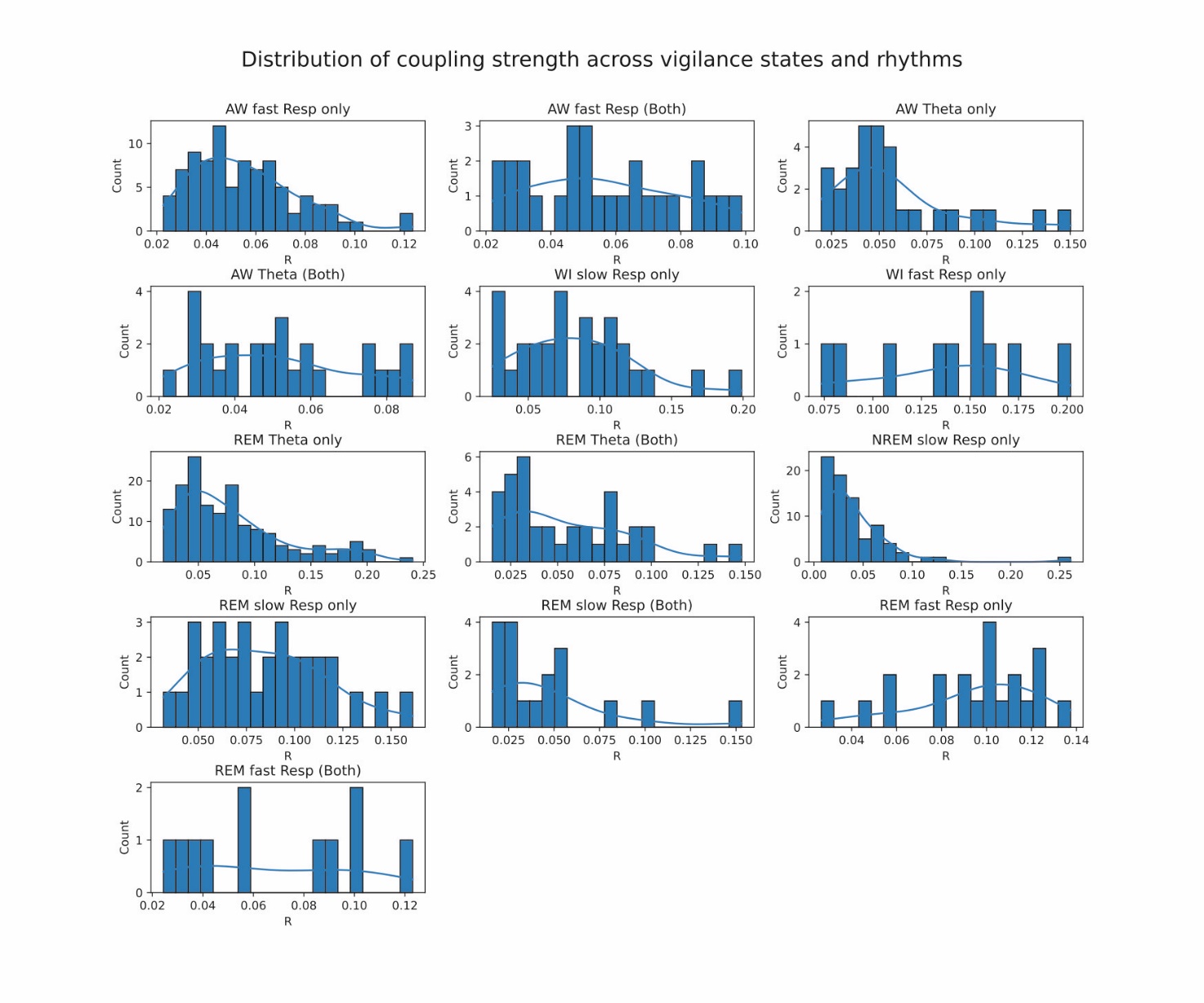
**

**Supplemental Figure 2: Distribution of coupling strength across units for vigilance states and slow oscillations.** Histograms depict the number of units significantly modulated by θ and/or Resp across vigilance states (blue line: smoothed kernel density estimate). Only: modulation by one rhythm (θ or Resp) but not the other; both: significant co-modulation by θ and Resp simultaneously.


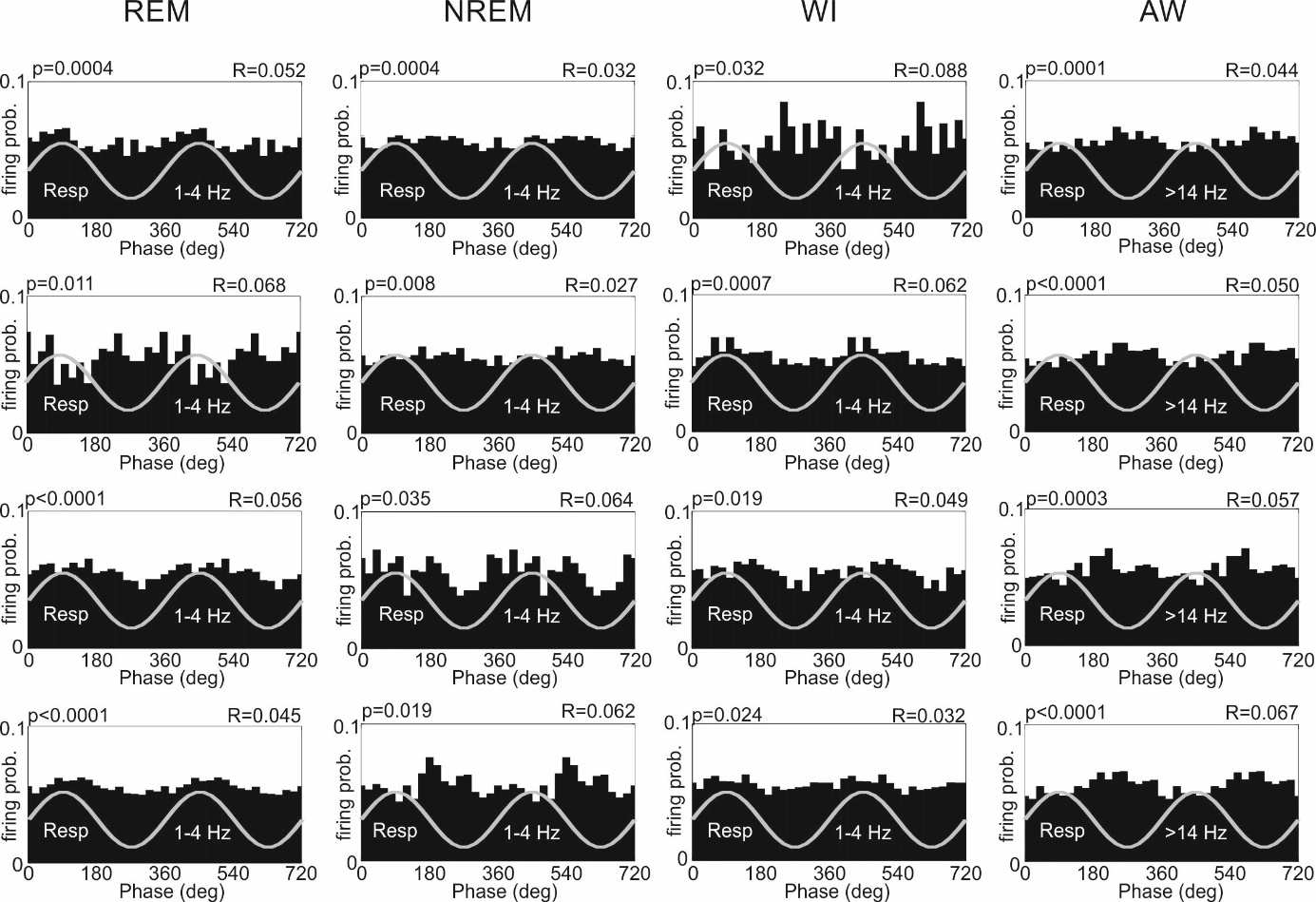


**Supplemental Figure 3: Examples of units coupled to respiration with medium coupling strength (R) in REM, NREM, WI and AW.**


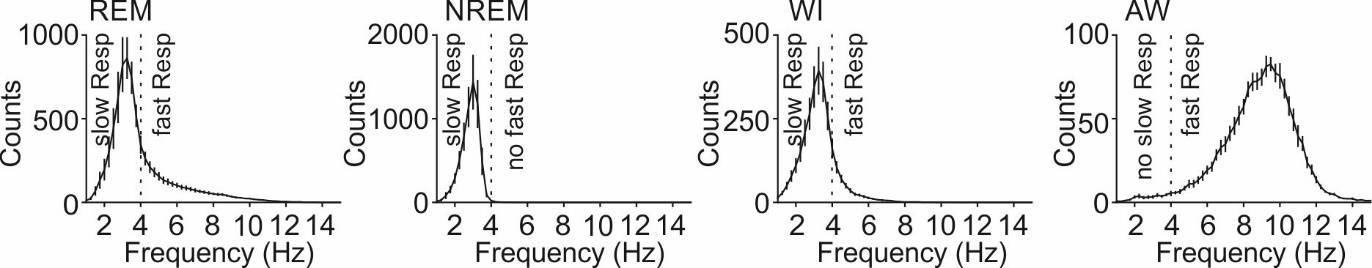


**Supplemental Figure 4: Mean histograms of respiration frequency counts in the four behavioral states.** Mean histograms of respiration (Resp) frequency (N=13 mice for REM, NREM, WI and N=10 mice for AW). Error bars indicate S.E.M.; vertical dotted line indicates threshold between slow Resp and fast Resp.

| **Supplemental Table 1. Interference of respiration and θ phases in both θ states** | | | | | | |
| --- | --- | --- | --- | --- | --- | --- |
| State | Depth (mm) | Unit type | N spikes | Mean angle | Comparison | U2 Watson |
| AW | 0-0.8 | θ only | 30 | 46.2 | θ vs. Both | p=0.0146 |
|  |  | Both (θ) | 27 | 16.2 |  |  |
|  | 0-0.4 | θ only | 21 | 69.2 | θ vs. Both | p=0.0612 |
|  |  | Both (θ) | 15 | 18.3 |  |  |
|  | >0.4 | θ only | 9 | 354.7 | θ vs. Both | p=0.5920 |
|  |  | Both (θ) | 12 | 13.9 |  |  |
|  | 0-0.8 | fR only | 89 | 192.3 | fR vs. Both | p=0.9679 |
|  |  | Both (fR) | 27 | 191.6 |  |  |
|  | 0-0.4 | fR only | 46 | 204.4 | fR vs. Both | p=0.7422 |
|  |  | Both (fR) | 15 | 194.3 |  |  |
|  | >0.4 | fR only | 43 | 180.9 | fR vs. Both | p=0.0156 |
|  |  | Both (fR) | 12 | 186.5 |  |  |
| REM | 0-0.8 | θ only | 154 | 168.7 | θ vs. Both | p=0.06270 |
|  |  | Both (θ) | 36 | 133.6 |  |  |
|  | 0-0.4 | θ only | 83 | 147.5 | θ vs. Both | p=0.2627 |
|  |  | Both (θ) | 23 | 279.0 |  |  |
|  | >0.4 | θ only | 71 | 223.6 | θ vs. Both | p=0.0314 |
|  |  | Both (θ) | 13 | 161.0 |  |  |
|  | 0-0.8 | s+fR only | 59 | 76 | s+fR vs. Both | p=0.1569 |
|  |  | Both (s+f) | 36 | 335.6 |  |  |
|  | 0-0.4 | s+fR only | 28 | 109.3 | s+fR vs. Both | p=0.3712 |
|  |  | Both (s+f) | 23 | 279.0 |  |  |
|  | >0.4 | s+fR only | 31 | 70.2 | s+fR vs. Both | p=0.4547 |
|  |  | Both (s+f) | 13 | 16.9 |  |  |
|  | 0-0.8 | sR only | 35 | 90.0 | sR vs. Both | p=0.0976 |
|  |  | Both (sR) | 18 | 8.0 |  |  |
|  | 0-0.4 | sR only | 15 | 198.1 | sR vs. Both | p=0.1201 |
|  |  | Both | 9 | 334.5 |  |  |
|  | >0.4 | sR only | 20 | 86.5 | sR vs. Both | p=0.5448 |
|  |  | Both | 9 | 37.6 |  |  |
|  | 0-0.8 | fR only | 24 | 26.5 | fR vs. Both | p=0.1082 |
|  |  | Both (fR) | 11 | 205.6 |  |  |
|  | 0-0.4 | fR only | 13 | 90.2 | fR vs. Both | p=0.0825 |
|  |  | Both (fR) | 9 | 202.2 |  |  |
|  | >0.4 | fR only | 11 | 348.6 | fR vs. Both | p=0.8808 |
|  |  | Both (fR) | 2 | 358.8 |  |  |
| fR: fast Resp (>4 Hz), sR: slow Resp (1-4 Hz), s+fR: slow and fast Resp (1-14 Hz) | | | | | | |

| **Supplemental Table 2. Depth differences** | | | | | | |
| --- | --- | --- | --- | --- | --- | --- |
| State | Depth (mm) | Unit type | N spikes | Mean angle | Comparison | U2 Watson |
| AW | 0-0.4 | θ only | 21 | 69.2 | 0-0.4 vs. >0.4 | p=0.2167 |
|  | >0.4 |  | 9 | 354.7 |  |  |
|  | 0-0.4 | Both (θ) | 15 | 194.3 | 0-0.4 vs. >0.4 | p=0.8083 |
|  | >0.4 |  | 12 | 186.5 |  |  |
|  | 0-0.4 | fR only | 46 | 204.4 | 0-0.4 vs. >0.4 | p=0.4166 |
|  | >0.4 |  | 43 | 180.9 |  |  |
|  | 0-0.4 | Both (fR) | 15 | 194.3 | 0-0.4 vs. >0.4 | p=0.0896 |
|  | >0.4 |  | 12 | 186.5 |  |  |
| WI | 0-0.4 | sR only | 6 | 101.4 | 0-0.4 vs. >0.4 | p=0.2819 |
|  | >0.4 |  | 23 | 49.9 |  |  |
|  | 0-0.4 | fR only | 5 | 297.2 | 0-0.4 vs. >0.4 | p=0.2577 |
|  | >0.4 |  | 5 | 334.7 |  |  |
| NREM | 0-0.4 | sR only | 42 | 208.4 | 0-0.4 vs. >0.4 | p=0.0519 |
|  | >0.4 |  | 36 | 216.1 |  |  |
| REM | 0-0.4 | θ only | 83 | 147.5 | 0-0.4 vs. >0.4 | p=0.0008 |
|  | >0.4 |  | 71 | 223.6 |  |  |
|  | 0-0.4 | Both (θ) | 23 | 116.9 | 0-0.4 vs. >0.4 | p=0.0376 |
|  | >0.4 |  | 13 | 161.0 |  |  |
|  | 0-0.4 | sR only | 15 | 198.1 | 0-0.4 vs. >0.4 | p=0.1025 |
|  | >0.4 |  | 20 | 86.5 |  |  |
|  | 0-0.4 | Both (sR) | 9 | 334.5 | 0-0.4 vs. >0.4 | p=0.2503 |
|  | >0.4 |  | 9 | 37.6 |  |  |
|  | 0-0.4 | fR only | 13 | 90.2 | 0-0.4 vs. >0.4 | p=0.5215 |
|  | >0.4 |  | 11 | 348.6 |  |  |
|  | 0-0.4 | Both (fR) | 9 | 202.2 | 0-0.4 vs. >0.4 | p=0.3516 |
|  | >0.4 |  | 2 | 358.8 |  |  |
| fR: fast Respiration (>4Hz), sR: slow Respiration (1-4 Hz) | | | | | | |
